# Cognitive dysfunction in diabetes: abnormal glucose metabolic regulation in the brain

**DOI:** 10.3389/fendo.2023.1192602

**Published:** 2023-06-16

**Authors:** Shan Zhang, Yueying Zhang, Zhige Wen, YaNan Yang, Tianjie Bu, Xiangwei Bu, Qing Ni

**Affiliations:** Department of Endocrinology, Guang’ anmen Hospital, China Academy of Chinese Medical Sciences, Beijing, China

**Keywords:** cerebral glucose metabolism, diabetes, cognitive dysfunction, insulin signal, molecular mechanism

## Abstract

Cognitive dysfunction is increasingly recognized as a complication and comorbidity of diabetes, supported by evidence of abnormal brain structure and function. Although few mechanistic metabolic studies have shown clear pathophysiological links between diabetes and cognitive dysfunction, there are several plausible ways in which this connection may occur. Since, brain functions require a constant supply of glucose as an energy source, the brain may be more susceptible to abnormalities in glucose metabolism. Glucose metabolic abnormalities under diabetic conditions may play an important role in cognitive dysfunction by affecting glucose transport and reducing glucose metabolism. These changes, along with oxidative stress, inflammation, mitochondrial dysfunction, and other factors, can affect synaptic transmission, neural plasticity, and ultimately lead to impaired neuronal and cognitive function. Insulin signal triggers intracellular signal transduction that regulates glucose transport and metabolism. Insulin resistance, one hallmark of diabetes, has also been linked with impaired cerebral glucose metabolism in the brain. In this review, we conclude that glucose metabolic abnormalities play a critical role in the pathophysiological alterations underlying diabetic cognitive dysfunction (DCD), which is associated with multiple pathogenic factors such as oxidative stress, mitochondrial dysfunction, inflammation, and others. Brain insulin resistance is highly emphasized and characterized as an important pathogenic mechanism in the DCD.

## 1 Introduction

Type 2 diabetes (T2DM) is a complex endocrine disease characterized by high blood glucose levels. Recently, cognitive dysfunction, encompassing cognitive decline, mild cognitive impairment (MCI) and dementia, has gained recognition as a significant comorbidity and complication of diabetes, which can impact an individual’s well-being and diabetes management (1). A study has revealed that individuals with diabetes experience cognitive dysfunction, which includes impairments in memory, reduced processing speed, and difficulties with executive function. These deficits are severe enough to interfere with daily activities and are observed across all age groups (2). Epidemiological studies have established that individuals with diabetes have an increased risk of dementia (1.6-fold) (3). Before the onset of dementia, patients with T2DM often suffer from mild to moderate cognitive impairment. Data has shown that the prevalence of MCI in T2DM patients is high worldwide (45%), especially in Asia, particularly China (46.4%). As of 2017, approximately 451 million people had diabetes, and this number is expected to increase to 693 million by 2045, with T2DM accounting for almost all (90-95%) cases (4). Considering the high incidence of the disease, there are currently no diabetes-specific medical therapies that have been proven to reduce the risk of cognitive dysfunction in diabetes. Thus, developing a better understanding of the mechanisms underlying diabetes is particularly essential for preventing or treating cognitive dysfunction associated with the disease.

Proper brain function and physiology are critically dependent on glucose as the brain’s primary energy source, highlighting the importance of regulating glucose metabolism. Recent studies have provided evidence that perturbations in cerebral glucose metabolism may be a significant contributor to the pathogenesis of diabetic cognitive dysfunction (DCD). Remarkably, these perturbations can occur even decades before the onset of cognitive dysfunction and pathological changes (5). Hyperglycemia and glucose metabolism disorders, commonly associated with diabetes, not only occur in peripheral tissues but also affect the brain (6, 7). When glucose metabolism is disturbed, the brain’s supply of glucose may be affected, which can have significant impacts on brain structure and function. Moreover, insulin signal is essential for the regulation of glucose transporter, glucose metabolism- associated genes/proteins and enzymes (8). Cerebral insulin resistance or deficiency induced by diabetes represents as an important pathomechanism that affects the regulation of energy source and brain function.

The aim of this review is to discuss the role of abnormal glucose transportation and intracellular glucose catabolism dysfunctions in the pathophysiology of DCD. We propose a hypothesis that impaired cerebral glucose metabolism induces and aggravates multiple pathogenic cascades, including activated oxidative stress, inflammation decreased neurotransmitter synthesis, and aberrant synaptic plasticity. These processes can lead to neuronal degeneration and ultimately cognitive deficits in DCD. Brain insulin resistance is highly emphasized as a major pathogenic mechanism in the disease.

## 2 The brain features of patients with DCD

### 2.1 Diabetic patients usually suffer from cognitive decrements and an increased risk of developing MCI or even dementia

In adults with T2DM, deficits in cognitive function can be divided into three stages according to severity: diabetes-associated cognitive decrements, MCI, and dementia (9). Diabetes-associated cognitive decrements refer to subtle changes in cognitive function. The subtle cognitive changes may be observed in one or several domains (10, 11). Palta et al. summarized studies that identified small to moderate cognitive decrements in T2DM patients relative to nondiabetic controls in six cognitive domains examined. Motor function demonstrated the largest effect size, following by executive function, processing speed, verbal memory, visual memory, while attention exhibited the smallest effect size (11). The cognitive decrements are likely to have an onset in the early stage of diabetes and last for years, at a rate that is up to twice as fast as that of people with normal cognitive (2, 12, 13). Moreover, this change occurs across all age stages in people with T2DM (13).

MCI has been identified as a prodromal stage of dementia, typically 1-1.5 SDs below normative data, affecting one or more cognitive domains with largely preserved activities of daily life. Several population-based prospective studies have reported on the risk of MCI in patients with T2DM. One study observed that T2DM patients had a hazard ratio (HR) of 1.58 (1.17-2.15) for amnesic MCI and 1.37 (0.84-2.24) for non-amnesic MCI during four years of follow up (14). Recent meta-analyses including nine studies also identified diabetes and its association with the risk of MCI, providing moderate quality evidence. Compared with non-diabetes, diabetes was associated with a 49% increased risk of MCI (RR:1.49, 1.26-1.77) (15). This stage of cognitive dysfunction might progress to dementia (9).

Dementia is defined as acquired objective cognitive impairment that affects several cognitive domains, severe enough to impact the activities of daily life. Meta-analyses, including over 144 prospective studies with over 9 million participants, estimated the RR in diabetes for all-cause dementia at 1.43 (1.33-15.3), for Alzheimer’s disease (AD) at 1.43 (1.25-1.62), and for vascular dementia at 1.91 (1.61-2.25) in individuals with diabetes (15). Abnormal fasting plasma glucose, 2hPG, HbA1c, and fasting plasma insulin (FPI) levels contribute to a higher risk of cognitive disorders. A meta-analysis during a median follow-up of 6.8 years showed that in participants with diabetes, higher glucose levels were accompanied by a higher HR for dementia, with a glucose level of 10.5 mmol/L as compared with 8.9 mmol/L, the adjusted HR was 1.4 (1.12-1.76) (16). Some studies showed that the risk of AD almost doubled in those with 2hPG of 7.8-11mmol/L and tripled in those with 2hG above 11.0 mmol/L compared to those with 2hPG below 6.7 mmol/L (17, 18). Diabetic patients with higher HbA1c variability were also associated with a higher risk of dementia, with HR:1.06 (1.003-1.120) (19). Evidence showed that higher levels of FPI were directly associated with dementia (RR:1.64, 1.33-2.02) (15). Moreover, glucose fluctuations also impact brain functional architecture and cognition in T2DM patients. T2DM patients with fluctuating glucose levels exhibited significantly worse performance on the Montreal Cognitive Assessment, Trail Making Test-B, and verbal fluency test, as well as significant decreases in degree centrality in some brain regions (20).

### 2.2 Morphological changes of brain with T2DM

Brain atrophy is defined as low brain volume that affects a specific brain region or tissue class, reflecting neuronal loss (21). Changes in cognitive function in patients with T2DM are often accompanied by structural brain abnormalities on magnetic resonance imaging (MRI). A study indicated that localized atrophy in the hippocampal area, a brain region critical to memory formation and consolidation, might be primarily responsible for the memory deficits seen in this population (22). Hisayama et al. investigated the association between diabetes and hippocampal atrophy in 1238 Japanese subjects who underwent MRI examination. The results showed that diabetic subjects, especially those with post-glucose-loaded hyperglycemia, had a significantly lower volume than those without diabetes. This Study also indicated that the hippocampal volume becomes smaller with longer duration of diabetes (23). In the Rotterdam scan study, people with T2DM had more hippocampal and amygdalar atrophy compared to nondiabetic subjects (24). Some study suggested that diabetes-related volumetric brain differences were substantial (total brain:1.88%; grey matter:2.81%; white matter:2.15%; hippocampus:4.4%), which began occurring in early adulthood. Further analysis showed these differences observed in T2DM correspond to about 4 to 5 years of normal ageing, and possibly more (25).

## 3 The features of brain glucose metabolism in diabetes

### 3.1 Hyperglycemia in the brain is an important feature of diabetes

Hyperglycemia is an important characteristic change caused by diabetes in the brain.

Brain glucose levels rise in a linear fashion with rising blood glucose levels rise. Evidence has shown a linear relationship between glucose levels in the cerebral and vascular compartments after stabilizing plasma glucoses in a range of 4–30 mM, with brain glucose levels being 20–30% of plasma levels (26). The plasma-to-brain glucose ratio appeared to follow the same trend in Wistar rats under normo- or hyper- glycemia (27). A study has compared the glucose level in brain extracellular fluid under a hyperglycemic state between diabetic and non-diabetic rats. High plasma glucose level (22 mM) led to elevated level of brain glucose (7.5 mM) in chronic diabetic groups, while the brain glucose in the normoglycemic rat brain was 2.1 mM at a plasma glucose level of 8 mM (6). Other studies performed on hyperglycemic patients also showed similar results. The patients showed brain glucose levels of 1.56 mM at a mean plasma glucose level of 11.5 mM, while the healthy control displayed a mean brain glucose level of 0.83 mM in a euglycemic state (28). These findings strongly demonstrate that hyperglycemia in the peripheral blood can directly result in brain hyperglycemia, which is an important feature in diabetes. Additionally, T2DM patients have a blunted rise in brain glucose levels. A study has shown that compared with lean participants without diabetes, brain glucose increments in response to a standardized increase in plasma glucose levels were lower in participants with poorly controlled T2DM, suggesting that chronic hyperglycemia is associated with blunted brain glucose transport and/or glucose metabolism, thus limiting glucose uptake as an adaptive mechanism (29). This may mean that long-term high blood glucose levels in the brain reduce the brain’s sensitivity to peripheral blood sugar responses.

### 3.2 Cerebral glucose hypometabolism is a biomarker of cognitive dysfunction in diabetes

An improved understanding of pathological regulation in glucose metabolism is impacting on researchers’ conception on diabetic cognitive dysfunction. The brain dominantly relies on glucose as the energy source, making it vulnerable to impaired energy metabolism. The use of neuroimaging technologies has allowed us to investigate the association between brain energy metabolism and DCD onset and progression. Positron emission computed tomography (PET) is an important tool for assessing glucose metabolism. When it is transported into brain cells, 2-Fluorine-18-Fluoro-2-deoxy-D-glucose (18F-FDG) can’t be catalyzed to glucose-6-phosphate (G6P) by glucose-phosphate-isomerase and is accumulated in the neurons as 18F-2-FDG-6-phosphate, which is a suitable radiotracer used to measure the local cerebral metabolic rate of glucose in the brain (30). Increasing evidence has shown that there is an apparent decrease of glucose metabolism in specific brain regions on Fludeoxyglucose F18-positron emission tomography (FDG-PET) scans in people with T2DM with cognitive impairment or not (31, 32). Different brain regions have varying susceptibilities to hypometabolism. Generally, hypometabolism has predilection sites for the frontal, temporal parietal, cingulate gyrus, and temporal gyrus regions (33, 34). In contrast, brain regions including the primary motor and visual cortex, cerebellum, thalamic and basal ganglia nuclei are rarely affected (35). Studies also indicated that the hippocampal region might be the first region of the brain affected by T2DM and that individuals with poor glucose metabolic control might be more obviously impacted (33). The reduction of local cerebral glucose metabolic rate (CMRglu) measured by 18F-FDG-PET also reflects the regional distribution of reduced synaptic density and activity in DCD diseases. The finding provided evidence that CMRglu was reduced in adults with prediabetes and early T2DM, involved the posterior cingulate cortex, the precuneus region, parietal cortices, the temporal/angular gyri, and the anterior and inferior prefrontal cortices. Compared to the normal individuals, these patients showed a different pattern during the memory encoding task, characterized by more diffuse and extensive activation, and recalled fewer items on the delayed memory test (5). A cross-sectional study showed that worse executive functions and memory were correlated with decreased gray matter density and low glucose metabolism in the orbital and prefrontal cortex, temporal cortex (middle gyrus, para hippocampus, and uncus), and cerebellar regions (36). Administration of the antidiabetic drug, Glucagon-like peptide 1, decreased intracerebral glucose content and raised cerebral metabolic rate in specific regions to markedly improve cognition and memory in diabetic patients (37).

Glucose hypometabolism also manifests in animals with T2DM. Streptozotocin (STZ)-induced diabetic mice with cognitive impairment had a peculiar metabolic phenotype-decrease energy metabolism in the brain regions (32). Soares et al. observed a tendency of reduced cerebral metabolic rate of glucose in T2DM rats, indicating impaired glucose utilization in T2DM (38). Like the human brain, brain region-specific metabolic disorders also exist in T2DM mice with cognitive impairment. Notably, metabolic patterns in hippocampus were largely differentiated in T2DM mice with cognitive impairment relative to control mice (38). Decreased glucose metabolism was found in diabetic mice’s brain, while increasing glucose availability in specific brain areas can positively modulate the performance in cognitive task in T2DM mice (39). Measuring other tracer uptake of radioactively labeled D-glucose, 2DOG, or 3OMG into the total brain or vascular endothelial cells (VECs), decreased glucose uptakes were observed in animals with diabetes (40, 41). In all, cerebral glucose hypometabolism can be considered as a biomarker in DCD. Glucose hypometabolism in specific brain regions contributes to DCD onset and progression. It indicates that improving hypoglycemic metabolism in the brain can inhibit/alleviate cognitive dysfunction in diabetes.

### 3.3 Impaired glycogen metabolism in T2DM

Impaired glycogen metabolism is another significant characteristic in the brain of individuals with T2DM. Glycogen is produced and stored exclusively in astrocytes, where they are concentrated near adjacent axons and dendritic spines favoring neurons (21). Astrocytic glycogen breakdown is essential for long-term memory formation and maintenance of long-term potentiation of synaptic strength (42). Memory consolidation is impaired when brain glycogen synthase is knocked out or glycogenolysis is inhibited before a memory-evoking event (43). Glycogen content were affected in various brain regions to varying degrees in diabetes. In rats with T2DM, reduced glycogen labeling was detected by^13^C magnetic resonance spectroscopy, with the most significant reduction in the hippocampus (33%) and hypothalamus (29%), following by striatum (25%), and cortex (24%) (44). Diabetic patients’ glycogen content in the occipital lobe was significantly reduced compared to healthy individuals (45). Evidence showed that downregulation and mislocalization of glucose transporter (GLUT) 4, and decreased glycogen storage in insulin-like growth factor (IGF) 1 null mice’ brain led to misutilization of neuronal glucose, impaired growth, and compromised brain functionality (42). The turnover rate of brain glycogen also strongly reflects impaired glycogen metabolism. Newly synthesized glycogen level in diabetic patients were lower than those in the control group, indicating reduced glutamine synthetase (GS) activity and glycogen turnover rate in the brain might be reduced (45). These data elucidate that hyperglycemia, decreased glucose utilization, and impaired glycogen metabolism are important cerebral features in diabetic state, reflecting severely dysregulated glucose metabolism in the brain. In the next section, we will introduce the specific mechanisms of impaired glucose metabolism in diabetic disease.

## 4 Impaired glucose transport or uptake

### 4.1 Abnormal glucose transport or uptake in the brain

Transporters were needed for the glucose entrance into the brain. Glucose transporters are 12-transmembrane glucose transport proteins that have been reported to have 14 isoforms (GLUT 1-14) (43). Within the brain, GLUT1, is expressed abundantly as a 55-KD isoform on the endothelium of the blood brain barrier (BBB), facilitating glucose transport from the circulation into the extracellular fluid. Meanwhile, a second 45-KD isoform of GLUT1 ensures delivery of glucose to glia, ependymal cells, and choroid plexus. Astrocytes express both GLUT1 and a specific GLUT2 isoform, while neurons predominantly express GLUT1 and GLUT3, the latter of which has a higher glucose affinity and transport capacity. While other GLUT family members are present in lower levels within the brain, their functions remain relatively understudied. Kinetic modeling predicted that glucose diffuses mostly *via* interstitial fluid, with glucose uptake and release by cells depending on local concentrations and supply-demand relationships (46, 47). Simpson et al. reported that GLUT1 transporter had a direct effect on rates of glucose uptake into the brain, with increased GLUT1 transporters supporting increased glucose uptake into the brain (48).

Many studies have shown that the expression, regulation, and the activity of GLUTs can be disrupted during hyperglycemia. These changes negatively affect glucose uptake and metabolism in the brain, resulting in impairment of synaptic plasticity, neurogenesis, and even cognitive function. Clinical studies revealed that the expression of GLUT1 and GLUT3 were decreased in the different brain areas in individuals with T2DM and AD, which may be attributed to (at least in part) the brain glucose hypometabolism (49). Study concluded that patients with T2DM have an increased risk of developing dementia through observing human brain samples (11 T2DM, 10 AD, 8 T2DM and AD, and 7 controls). It showed that T2DM brain had a decreased level of neuronal GLUT3 protein compared with the AD brain and that the decreased O-GlcNAcylation (inhibiting tau protein) observed in the AD brain was also visible in the T2DM (50). In parallel with the decrease in HbA1C, the improvement of brain glucose utilization was observed in the brain regions of T2DM, highlighting the potential reversibility of cerebral glucose transport capacity and metabolism that can occur in individuals with T2DM following improvement of glycemic control (51). Some researchers considered that glucose excess in the extracellular space in the brain, causing a downregulation of GLUT1 and GLUT3 proteins. These low expressed transporters might be the body’s adaptive response to prevent excessive glucose entering the cell which can lead to cellular damage (52). Whatever the explanation, cerebral hyperglycemia is a major factor responsible for the abnormal glucose transport or uptake in diabetes.

The high fat diet (HFD) mouse is a widely used model for studying insulin-resistant in diabetes. Brain glucose uptake and GLUT1 in the VECs were reduced in the HFD feeding animals, exhibiting impaired learning and memory (53, 54). An *in vivo* experiment demonstrated that VECs exposed to high glucose levels downregulated the rate of glucose transport by decreasing GLUT1 mRNA and protein, as well as the localization of GLUT1 to the plasma membrane (55). GLUT1, GLUT3, and GLUT4 were significantly decreased in the brain of diabetic animals by 61%, 69% and 64%, respectively (56). GLUT1 mRNA and protein in hippocampus were significantly decreased (57). In neurohypophysis of rats with STZ-induced diabetes, GLUT1 was decreased 16% by 3 days and 25% by 1-2 week of diabetes (58). Cardoso reported that long-term hyperglycemia down-regulated GLUT3 protein levels in brain cortical tissue of diabetic rats (59). Impaired GLUTs can set off a cascade of abnormal reactions including BBB breakdown, accelerated amyloid β -peptide (Aβ) pathology, reduced Aβ clearance, diminished neuronal activity, progressive neuronal loss, ultimately causing behavioral deficits and cognitive dysfunction (60). On the contrary, improving GLUT utilization and glucose uptake may benefit cerebral glucose metabolism, which relieves cognitive dysfunctions in diabetes. This point has been proved in some studies. Central injection of liraglutide (an antidiabetic drug) improved memory functions in diabetic rats, which correlated with the ameliorated brain glucose uptake (61). Overexpression of a glucose transporter or stimulation of glucose uptake by metformin attenuated cognitive dysfunction in T2DM, suggesting that the recovery of glucose transport and uptake activities are beneficial to the neuronal function repair (62).

### 4.2 Disrupted Insulin signal with GLUT transport

The mechanism by which GLUT transporters decrease in the brain in diabetes is not well understood. However, studies have shown that insulin and IGF1 activate signal transduction in cells to mobilize GLUTs to the cell membrane, which facilitate glucose entry into neuronal cells (63, 64). In diabetes, insulin resistance involving the dis-regulated phosphatidylinositol 3 kinase (PI3K)/protein kinase B (AKT) pathway is a common characteristic. T2DM with cognitive impairment has been linked to decreased levels of insulin receptor substrate (IRS)1, PI3K, and AKT proteins (65–67). An autopsy study has shown that the brain insulin/PI3K/AKT signaling pathway is impaired in T2DM and AD patients, with greater impairment in AD-T2DM patients (68). Griffith et al. reported GLUT3 translocation was observed in the hippocampus of mice with altered cognition due to decreased insulin levels (69). Down-regulation and mis-localization of GLUT4 were found to impair brain function in IGF1 null mice (42). Ablation of insulin receptor (IR) in astrocytes decreased brain glucose uptake (29). Improving insulin resistance is beneficial for the recovery of GLUT transport function. Intranasal insulin has been shown to bypass the BBB, target the brain, and improve synaptogenesis in rodent models, as well as memory in adult humans with T2DM or AD (70). In contrast, decrease in tyrosine phosphorylation of IR and an increase in serine phosphorylation of IRS1 lead to PI3K/AKT dysregulation and glucose uptake reduction in HFD induced mice (71, 72). Overall, deficiency in GLUT transporter proteins and activity can have a significant impact on brain glucose energy metabolism, and the insulin pathway plays a critical role in GLUTs expression and function. Regulating the insulin signal and enhancing the expression of GLUTs could be a novel strategy for reversing cognitive dysfunction in diabetes.

## 5 Intracellular glucose catabolism dysfunctions

### 5.1 Worse glycolysis in the brain

Glucose taken up by cells can be channeled into glycolysis pathway leading to the formation of pyruvate, or shunted to the nicotinamide adenine dinucleotide phosphate (NADPH)-producing pentose phosphate pathway (PPP) pathway. Research suggests that impaired glycolytic function may contribute to the pathophysiology of diabetes-related cognitive impairment. A study found 19 discriminating metabolites in the brain of individuals with DCD compared to controls, indicating potential impairment in the glycolysis process (73). Similarly, a study in DCD mice found significant differences in glycolysis intermediates, such as hexose bisphosphate, in the brain (74). Dysregulated glycolysis resulting in lactate accumulates is a common feature of the DCD brain and may hinder pyruvate’s entry into the tricarboxylic acid (TCA) cycle. For example, hyperpolarized [1-13C] pyruvate MR spectroscopy was used to detect lactate content in a HFD-induced cognitively impaired mouse model, showing that the conversion of pyruvate to lactate were significantly increased in different brain regions, especially in the hippocampus. This findings suggested that increased lactate levels may be a virtual pathogenic factor in DCD (75). Another study also found that diabetes induced a significant increase in lactate level in the hippocampus, striatum, hypothalamus, and midbrain of mice (76, 77).

We next discussed the dysregulation of glycolytic enzymes and metabolites in the brain of diabetic mice. Hexokinase (HK) activity significantly increased by 14% in the hypothalamus and 15.5% in the tractus solitarius. Substrate kinetic properties of key glycolytic enzymes including HK, phosphofructokinase (PFK) 1, pyruvate kinase (PK), were affected by recurrent hypoglycemia exposure in the hippocampus of diabetic animals (78). Pyruvate dehydrogenase kinase isoform 2 (PDK2) inhibits the pyruvate dehydrogenase complex by phosphorylating and inactivating one of its subunits, pyruvate dehydrogenase (PDH), thereby preventing the conversion of pyruvate to acetyl coenzyme A (acetyl-CoA), which then enters the TCA cycle for further energy production. Rahman et al. found that diabetes in mice enhanced the hypothalamic expression of PDK2 and phosphorylated-PDH, causing a glycolytic metabolic shift along with substantial hypothalamic inflammation. Genetic ablation or hypothalamic inhibition of PDK2 attenuated diabetes-induced neuroinflammation, lactate surge in the hypothalamus in mice (79). Pyruvate dehydrogenase E1 alpha subunit (PDHA1) is the core component of the pyruvate dehydrogenase complex. PDHA1 knockout damaged the spatial memory of mice and led to the ultrastructural disorder of hippocampal neurons (80). Lactate accumulation and abnormal lactate transport were also found in PDHA1^-/-^ mice. Lactate dehydrogenase (LDH), which catalyzed the conversion of pyruvate to lactate, was also markedly upregulated, indicating increased glycolytic activity (81, 82). Overall, these findings suggest that dysregulation of enzymes in the glycolytic process were observed, resulting in lactate accumulation, which is a common feature of diabetes-related cognitive decline.

### 5.2 Abnormal insulin pathway with glycolysis

A role for insulin signaling has been implicated in this regard. Insulin negatively regulates Forkhead transcription factors (FOX) families through the PI3K/AKT signal such as FOXO1, FOXK1 (83, 84). These proteins are tightly associated with the related protein expressions of glycolysis, such as HK2, PFK, PKM, LDH, and PDH (85). Extensive studies in diabetes over the past decade have shown that FOX factors are highly expressed in the brain, which are likely to be mediated by decreased sensitivity to insulin (86–88). FOXO1 enters the cell nucleus and promotes the activities or/expressions of HK and G-6-pase (86). FOXK1 induces aerobic glycolysis by upregulating the enzymatic activities (HK-2, PFK, PK, and LDH), while at the same time suppressing further oxidation of pyruvate in the mitochondria by increasing the activity of PDH1 and PDH4 (88). Mitogen-active protein kinase (AMPK) is the cellular energy sensor that acts to restore and maintain the energy balance within the cell. AMPK up-regulates insulin sensitivity. In DCD models, AMPK was inhibited in the hippocampus of the brain (89). Moreover, Activation of Wnt Signal in cortical neurons enhances glucose utilization through glycolysis and thus plays a neuroprotective role (90). However, the effect of Wnt signal is partially abolished accompanied by dis-regulated glycolysis. Insulin could attenuate the neuronal damage and cognitive dysfunction in diabetic mice by enhancing the Wnt pathway (61). Therefore, the neuroprotective effects of insulin signaling in DCD mouse models can be attributed, at least in part, to insulin signal-mediated improvement in glucose metabolism and utilization in the glycolysis process.

### 5.3 Abnormal regulation in PPP

The PPP branches utilize G6P to produce fructose 6-phosphate and glyceraldehyde 3-phosphate through both oxidative and non-oxidative pathways, thereby supplying ribose 5-phosphate and NADPH. The conversion of oxidized glutathione to reduced glutathione is a crucial step in the cellular response to oxidative stress, and this process depends on NADPH through the action of glutathione reductase. NADPH is also a substrate for NADPH oxidases (NOXs), generating reactive oxygen species (ROS). Under normal physiological conditions, the harmfully species is counterbalanced by antioxidant systems. However, in some cases, this balance is disrupted, leading to oxidative stress. Spatially resolved metabolic profiling of the brain in diabetic models has revealed increased PPP activity (73), likely due to alterations in brain NADPH metabolism caused by hyperglycemia. Glucose-6-phosphate dehydrogenase (G6PD) and 6-phosphogluconate dehydrogenase (6PGD) are the rate-limiting enzyme in PPP pathway and produce NADPH in cells. In diabetic rat brains, G6PD was markedly increased compared to controls (91). Neonatal hyperglycemic rats presented increased activities of G6PD, 6PGD, and NOXs, which might be responsible for the enhanced superoxide radical anion production. Although enhanced antioxidant enzyme activities (SOD, Catalase, and glutathione peroxidase) were observed in hyperglycemic rats, they were unable to hinder the lipid peroxidation and protein damage in the brain (92). Singh reported G6PD might be considered a biomarker of oxidative stress and poor glycemic control in diabetic patients (93). The significance of perturbations in the PPP pathway in the development of diabetes or as a consequence of the metabolic abnormalities associated with the disease remains unclear. Nevertheless, it is evident that abnormalities in the PPP pathway are prevalent in diabetes and may play a crucial role in the disease’s pathogenesis.

### 5.4 Dis-regulated insulin signal with PPP

Altered G6PD and G6PD activities in PPP are associated with insulin resistance in peripheral tissues and there is relatively little research on their relationships in the brain. Previous studies have showed that only optimal levels of G6PD and 6PGD are beneficial to insulin secretion. Patients with G6PD deficiency showed lower levels of insulin secretion (94). Suppression of G6PD and 6PGD by high glucose not only blocked the insulin secretion, but also increased oxidative stress response (95, 96). However, the overexpression of G6PD also negatively affected insulin secretion in the pancreas due to the increased accumulation of NOXs and ROS (97). Excessive ROS production can disrupt cellular homeostasis and alter intracellular signaling pathways, ultimately exacerbating insulin resistance. Some studies have reported that ROS reprogram glucose metabolism by up-regulating the ppp pathway and inducing insulin resistance in T2DM (98). A recent study has shown that hyperglycemia changed G6PD activity through the oxidative stress in the hippocampus, leading to the neuronal injury, and cognitive impairment in STZ-induced diabetic mice (99). However, the direct mechanisms between PPP and insulin signal in the brain require further elucidation.

### 5.5 Defective TCA cycle and oxidative phosphorylation process

The dysfunction of intracellular oxidative catabolism, including OXPHOS and TCA cycle in mitochondria, can affect cerebral glucose metabolism, which is likely to contribute to DCD. Pyruvate is converted to acetyl-CoA by the PDH, then the later enters the TCA cycle for mitochondrial oxidation. Previous study found that the deficiency of PDH led to different degrees of cognitive impairment, suggesting a serious alteration of cerebral glucose metabolism in DCD diseases (68). Furthermore, a decreased TCA cycle generating ATP was observed in hippocampus, the cortex and striatum of diabetic mice with cognitive impairment (75, 76). One of reasons can be attributed to the decreased protein levels of oxidative phosphorylation components, such as complex III (electron transport chain component) and ATP synthase, resulting in the production of less ATP (77). Similarly, decreased activities of complexes I and II of the mitochondrial respiratory chain have been observed in the hippocampus and prefrontal cortex of diabetic animals, and these changes could lead to mitochondrial impairment and neuroinflammation (100). Several TCA cycle intermediates, including succinate and citrate, were dis-regulated in the hippocampus of diabetic mice with cognition decline (101). Other studies also account for the decrement of ATP generation in DCD disease. Under hyperglycemia, more pyruvate is available to be oxidized in the TCA, thus increasing the flux of NADH and flavin adenine dinucleotide into the electron transport chain. This effect increases the voltage gradient across the mitochondrial membrane, reaching a critical threshold that blocks electron transfer inside complex III, causing the electrons to back up to coenzyme Q, which donates the electrons one at a time to molecular oxygen, thereby generating more superoxide when compared to cell under normal glycemia. These super oxides may cause neurons impaired (102). Notably, due to the abnormal glycolytic capacity and high energetic needs, neuronal cells are extremely dependent on mitochondria and, therefore, critically sensitive to mitochondrial alterations in structure, localization, and function. Studies have shown that increased OXPHOs function, improving glucose utilization, could prevent neuronal loss, and preserve cognition in diabetic rats (91). Any impairment in brain mitochondria electron chain may induce subsequent mitochondrial dysfunction and oxidative stress. In turn, the drastic oxidative stress may trigger apoptosis in neuronal cells, which represents a primary cause of the oxidative imbalance observed in DCD (103).

### 5.6 Impaired insulin signal with OXPHOS and TCA cycle

Insulin plays a crucial role in regulating mitochondrial function in the brain, affecting OXPHOS and TCA processes. Studies have demonstrated that administering insulin to healthy mice increased mitochondrial ATP production, highlighting its direct regulatory role in brain mitochondrial function (104). Kleinridders et al. showed that brain specific knock out of insulin receptor mice exhibited reduced mitochondrial oxidative activities (complex I-V) and damaged dopaminergic neurons (105). Blocking IR by S961 (an IR antagonist) also decreased ATP production in primary cortical neurons and astrocytes and led to increased accumulation of harmful ROS (106). Excess ROS further caused oxidative stress, exacerbated neuroinflammation, and induced cognitive damage in the brain of diabetic animals (102). Insulin also associates with brain mitochondrial structures and functions. Peroxisome proliferator-activated receptor gamma coactivator 1α (PGC-1α) is mainly involved in mitochondrial biogenesis, which strongly relates to neurodegenerative disorders. IRS1/2 knock out mice induced insulin resistance, decreasing FOXO1 suppression and leading to hyper-active FOXO1. This, in turn, damages the mitochondrial respiratory chain, reduces the NAD+ level, weakens NAD+-dependent PGC-1α deacetylation, and ultimately leads to decreased ATP production. Abi-Saab reported that insulin deprivation suppressed the mitochondrial fusion proteins (MFN1, MFN2, and OPA1) and increased fission protein (dynamin-related protein-1, DRP1), suggesting that insulin deficiency adversely affects mitochondrial dynamics (28). Evidence showed that DRP1, via a glycogen synthase kinase 3/DRP1 dependent pathway, damaged the mitochondrial morphology, impaired the activity of complex I, and prevented the ATP reduction in hippocampal neurons from insulin-resistant mice (107). As is well known, only ample energy produced by mitochondria could ensure synaptic plasticity and the efficiency of signal transmission. However, in diabetes, the decreased ATP production in the brain is insufficient to meet the energy demand of neurons, and the following consequence is neuronal dysfunction and death (102, 108). Disruption of insulin action in the brain leads to impairment of mitochondrial energy metabolism and neuronal function. This may partly explain why diabetic patients or models are susceptible to dementia. These data links altered insulin sensitivity in the brain and dysfunctional mitochondria to neurodegeneration.

### 5.7 Abnormal glucose metabolism “cross talk” between astrocyte and neuron

The neuronal cells have “cross talk” in the brain, especially between astrocyte and neuron. Astrocytes release lactate through the low-affinity peri synaptic monocarboxylate transporter (MCT) 1 and MCT4, which is then taken up by the high-affinity neuronal MCT2 and transformed into ATP in neurons. Glycogen is produced and stored exclusively in astrocytes and converted into lactate to supply neurons. It is important to note that lactate derived from glycogen in astrocytes plays a role in stimulating neuronal plasticity and learning (11, 18). Studies demonstrated the increased lactate accumulated in the different brain origins, implying dysregulated lactate shuttle between astrocyte and neuron (109). High extracellular lactate may cause neurotoxicity if neurons cannot efficiently utilize the lactate being produced by astrocytes, i.e. uncoupling of neuronal and astrocyte metabolism. Additionally, decreased expression of lactate transporters, including MCT1 and MCT4 in astrocytes, and MCT2 in neurons have been reported in T2DM animals with cognitive decline in some studies (110–112). Brain glycogen levels in different brain origins are also affected in animal models of T2DM in different directions, impacting energy and neurotransmitter metabolism in the brain (113). Apart from lactate, other metabolites shuttle between neurons and astrocytes. Glutamine can be synthesized by GS in astrocytes and transferred to neurons. In neurons, it can be catalyzed by glutaminase (GLS) to form glutamate, which is an excitatory neurotransmitter. Glutamate can be further metabolized by glutamate decarboxylase (GAD) to form gamma-aminobutyric acid (GABA), an inhibitory neurotransmitter in the central nervous system (16). This cycle is named glutamate/gabba aminobutyric acid-glutamine (Glu/GABA-Gln) cycle. Accumulation of the glutamine in the brain of diabetic rats indicates dysregulation of the Glu/GABA-Gln cycle, which leads to cognitive decline in diabetes mellitus (73). Elevated glutamine level and decreased levels of glutamate and GABA were observed in diabetic mice with cognitive impairment. Further analysis revealed that GS was increased, GLS and GAD were decreased in the brain of diabetic mice (101). Using 3C labeling incorporation, the study showed that diabetic rats displayed lower rates of brain GS, Glu/Gln cycle, and TCA cycle rate in neurons. In contrast, the TCA cycle rate of astrocytes was larger in diabetic rats than controls, also suggesting impaired brain energy metabolism between neurons and astrocytes (76). Based on these results, the balanced “cross talk” between astrocyte and neuron eventually leads to the reduced energy for neurons or even damages neuron functions, which plays an key role in many physiological processes in the brain, especially in DCD. Therefore, in the next paragraph, we would discuss the major adverse effects of changed glucose metabolism on nerve cells.

## 6 The adverse consequences caused by changed glucose metabolism

### 6.1 Reduced synthesis of neurotransmitters and neurotransmitter modulators

The synthesis of neurotransmitters and their modulators in the brain is closely linked to glucose metabolism. Glucose serves as a carbon and hydrogen carrier that participates in the synthesis of neurotransmitters and neuromodulators, such as acetylcholine, GABA, glutamate, glycine, serine, tryptophan, and others. It has been verified by tracking the carbon or hydrogen atoms transferred from glucose to other materials (114). Recent studies have highlighted the critical role of glucose metabolism in the production of neurotransmitters and neuromodulators. When glucose metabolism is impaired, the synthesis of these molecules can be disrupted, which in turn can contribute to neuronal dysfunction. For example, there were downward shifts in the levels of neurotransmitters (glutamate, glutamine, aspartate), amino acids (valine, leucine, isoleucine, taurine, succinate, glutathione, choline, glycine), and energy metabolites (ATP, ADP, AMP) in the 9th week of disease progression in diabetic mice, at which point significant pathological damage in the hippocampal region had already occurred. Notably, the levels of lactate and glucose increased throughout the hyperglycemic period (115). L-Alanine, L-Glutamine, L-Lysine, L-Serine, and L-Threonine were also identified as potential biomarkers for DCD. These biomarkers are mainly involved in glycine, serine, and threonine metabolism, alanine, aspartate, and glutamate metabolism, as well as glyoxylate and dicarboxylate metabolism, which are associated with TCA cycle (116). Acetylcholine is essential for integrating learning and memory functions. It is produced from choline and acetyl-CoA. A non-targeted metabolomics approach used in diabetic rats with cognitive impairment showed that choline metabolism was down-regulated compared with control (117). Valine, leucine, and isoleucine degradation, tryptophan metabolism, phenylalanine metabolism, glycine, serine and threonine metabolism, and phenylalanine, tyrosine, and tryptophan biosynthesis, etc. showed marked perturbations over diabetic mild cognitive impairment and may contribute to the development of disease (38, 118). Diabetes also disrupts the Glu/GABA-Gln cycle in the brain. As we mentioned in the previous paragraphs, impaired glucose oxidation inhibits the production of glutamate and GABA, and causes altered neuronal network activity (115, 119). L-serine is synthesized from the glycolytic intermediate 3-phosphoglycerate and converted to D-serine by serine racemase in neuronal cells (13). The latter is an NMDA-receptor agonist, plays a key role to influence synaptic plasticity (14). Deficits of L-serine caused by abnormal glycolysis progression in astrocytes have been shown to induce severe cognitive deficits (15, 16). These studies demonstrated the neurotransmitters and neurotransmitter modulators were impaired in glucose metabolism.

### 6.2 Aberrant synaptic plasticity

Importantly, the reduced ATP production brings serious consequences, inducing aberrant synaptic plasticity and promoting cognitive damage in diabetes. Primarily, decreased ATP in neurons results in a diminished ability to maintain ionic gradients, hindering production and propagation of action potentials and therefore neurotransmission. The imbalanced ionic gradient allows extracellular Ca^2+^ enter and raises Ca^2+^ concentration in the intracellular space, stimulating Ca^2+^ dependent endonuclease, phospholipase, and proteinase activities, leading to synaptic dysfunction and eventual neuronal death (120). Accumulated Ca^2+^ exceeds the regulatory capacity of the endoplasmic reticulum and mitochondria, consequently leading to the release of cytochrome c and apoptosis factor and provoking neuronal apoptosis (121). Excess free Ca^2+^ in neuron also causes a loss of fidelity of microtubule assembly, damaging neuronal structures and functions. What’s more, ATP has properties of a biological hydrotrope, which can prevent the formation of, or dissolve misfolded protein aggregates (122). For impaired neurons, ATP’s hydrotropic effect may enhance the solubility and clearance of toxic aggregates. Unfortunately, this ability of ATP has been inhibited in cognitive impairment of diabetes (123). Furthermore, some intermediates such as elevated ROS or lactate, may exacerbate synaptic plasticity, either directly or indirectly (81, 124). So far, impaired neuronal structure and synaptic plasticity have identified as a significant pathological mechanism of cognitive impairment (125). In views of this, decreased ATP availability in neurons may lead to structural and functional abnormalities in the neuronal cells, causing a “storm” of detrimental cognitive effects in the brain of DCD.

### 6.3 Contribution to oxidative damage and inflammation

Recent studies have shown that the impaired glucose metabolic pathway may enhance the oxidative stress and inflammation. A study showed that dysregulation of G6PD activity can lead to a reduction in NADPH, promoting oxidative stress, as we mentioned in the previous paragraph (126). ROS is produced within the cell by mitochondria. Lower levels of ROS in cells are fundamental signal molecules for physiological processes, such as redox homeostasis and signal transduction. However, several defects in mitochondrial function may result in ROS excessive production. Under the state of hyperglycemia, mitochondrial dysfunction with subsequent elevated ROS levels becomes detrimental for normal cellular signaling and causes an amplifying cascade of oxidative stress (127). During OXPHOS, H2O2 and O2^–^ are produced as byproducts in mitochondria, primarily by complexes I (NADH dehydrogenase) and III, and are sequestered by the antioxidant enzymes. However, under disease conditions, this balance may be altered, leading to excessive ROS production and cellular damage (128). This oxidative stress becomes an initiator of various pathological effects, especially inflammation and apoptosis, through different pathways and interweave into complex networks, finally damage various proteins related to glucose metabolism, and inducing the development and progression of diabetes in the brain (129, 130).

Microglia account for 10-15% of cells in the brain and play an important role in immune response and neuroplasticity (9). Under physiological conditions, microglia maintain a resting state characterized by their ramified morphology. When stimulated, microglia activate and undergo changes in morphology, proliferation, and release of cytokines as a response to immune actions. Highly activated hypertrophied microglial cells have been clearly observed in hippocampus during the occurrence of diabetes (131). Reportedly, oxidative stress activates the cell death process and recruits microglia to the damaging site, which then over-produces proinflammatory cytokines and neurotoxic mediators, aggerating the inflammation. Microglial energy metabolism is tightly associated with its activity. Evidence suggested that microglia increased glycolysis and decreased oxidative phosphorylation when activated by various stimuli (132). During inflammation in the brain, the high level of glycolysis in active microglia not only supports the production of inflammatory mediators but also consumes a vast amount of glucose that is desperately needed by neurons. Some studies have also demonstrated that the cerebral glucose uptake in brain regions of individuals is strongly influenced by microglial activity. Microglial activation states drive glucose uptake in AD disease patients and mice models (133). Therefore, further understanding the metabolic pathways of active microglia will be important to better control neuroinflammation and improve the management of neurodegeneration. The inefficient glucose metabolism pathway and oxidative stress/inflammation are intimately related. A major contributor of the former may well enhance oxidative stress, which almost always leads to impaired enzymic activities involved in glucose metabolism, thus creating a vicious circle.

In summary, cognitive dysfunction that is associated with diabetes is seemingly a consequence of neuronal damage. Diabetes-induced brain hyperglycemia and glucose metabolic disorders are closely intertwined with oxidative stress, inflammation, and other factors, which mutually promote and affect each other, leading to disruptions in the synthesis of essential amino acids and neurotransmitters in the brain, damage to neural structures and plasticity, and neurological function.

## 7 Conclusions

**Figure 1:**
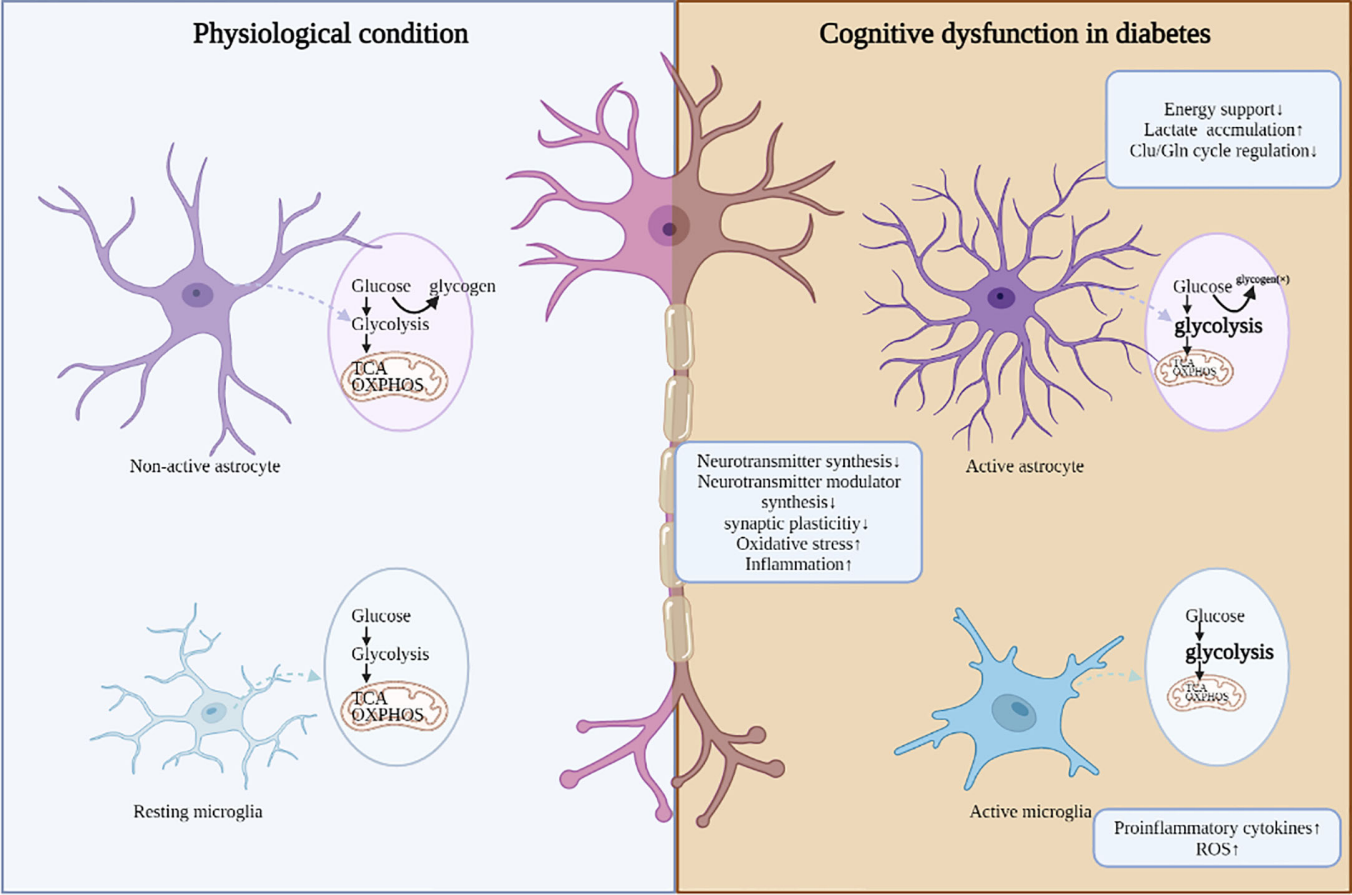
The adverse consequences induced by dis-regulated glucose metabolism in DCD. Under diabetic conditions, aberrant glucose metabolism in different cell types is accompanied with abnormal neuronal function, mainly including decreased synthesis of neurotransmitters and neurotransmitter modulator, aberrant synaptic plasticity, aggerated oxidative stress and inflammation.

The impact of hyperglycemia on the central nervous system has been the subject of increasing interest. Based on the discussions above, this review highlights the role of impaired cerebral glucose metabolism in the pathophysiological cascades of diabetes. Glucose transport, glycolysis, PPP, and TCA cycle in glucose metabolism are aberrant in the brains of diabetic patients and animals, leading to reduced ATP synthesis, aggerated oxidative stress/inflammation, all of which drive decreased synthesis of neurotransmitters and neurotransmitter modulator, aberrant synaptic plasticity, and ultimately neuronal damage and cognitive impairment (**Figure 1**). Among these pathogenic processes, impaired cerebral insulin signal is highly emphasized and characterized as an important pathogenic mechanism in the regulation of glucose metabolism. In future work, targeting abnormal cerebral glucose metabolism, such as improving brain insulin resistance, may be a promising strategy for improving diabetic cognitive dysfunction.

## Author contributions

SZ wrote the manuscript, YZ and ZW draw the figures, YY, TB and XB, searched the literature and edited the manuscript, QN reviewed and edited the manuscript. All authors contributed to the article and approved the submitted version.

## Glossary

**Table:**

6PGD	6-phosphogluconate dehydrogenase
Aβ	amyloid β -peptide
acetyl-CoA	acetyl coenzyme A
AD	Alzheimer’s disease
AMPK	mitogen-active protein kinase
BBB	blood brain barrier
DCD	diabetic cognitive dysfunction
DRP1	dynamin-related protein-1
FDG-PET	Fludeoxyglucose F18-positron emission tomography
FOX	Forkhead transcription factors
FOXO1	Forkhead box O1
FPI	Fasting plasma insulin
G6P	glucose-6-phosphate
G6PD	Glucose 6-phosphate dehydrogenase
GABA	gamma-aminobutyric acid
GAD	glutamate decarboxylase
Gln	glutamine
Glu	glutamate
Glu/GABA-Gln cycle	glutamate/gabba aminobutyric acid-glutamine
GLUTs	glucose transporters
GLS	glutaminase
GS	glutamine synthetase
HFD	high fat diet
HK	hexokinase
IGF	insulin-like growth factor
IR	insulin receptor
IRS	insulin receptor substrate
LDH	lactate dehydrogenase
MCI	mild cognitive impairment
MCT	monocarboxylate transporter
MRI	magnetic resonance imaging
NADPH	Nicotinamide adenine dinucleotide phosphate
NOXs	NADPH oxidases
OXPHOs	oxidative phosphorylation
PDH	pyruvate dehydrogenase
PDHA1	Pyruvate dehydrogenase E1 alpha subunit
PET	Positron emission computed tomography
PFK	phosphofructokinase
PGC-1α	Peroxisome proliferator-activated receptor gamma coactivator 1α
PI3K	phosphatidylinositol 3 kinase
AKT	protein kinase B
PK	pyruvate kinase
PPP	pentose phosphate pathway
ROS	reactive oxygen species
STZ	Streptozotocin
T2DM	type 2 diabetes
TCA	tricarboxylic acid
VECs	vascular endothelial cells
